# Urinary incontinence severity: the impact on workplace productivity

**DOI:** 10.1007/s00345-025-05822-y

**Published:** 2025-07-12

**Authors:** Marta G. Porto, João Marôco, Teresa Mascarenhas, Patrícia Vergamota, Inês Queiroz-Garcia, Filipa Pimenta

**Affiliations:** 1 William James Center for Research, Ispa – Instituto Universitário, Rua Jardim do Tabaco, 34, 1149-041 Lisbon, Portugal; 2 Ispa – Instituto Universitário, Lisbon, Portugal; 3 INTREPID LAB, ECEO, Universidade Lusófona & CETRAD, Lisboa, Portugal; 4 Department of Obstetrics and Gynecology, Faculty of Medicine, CHSJ-EPE, University of Porto, Porto, Portugal

**Keywords:** Urinary symptoms, Symptom burden, Occupational productivity, Help-seeking behavior

## Abstract

**Purpose:**

Urinary Incontinence (UI) is a highly prevalent yet underreported condition among middle-aged women, with its symptom severity significantly impacting Workplace Productivity (WP). This study aimed to explore the impact of UI symptom severity on the WP of middle-aged women and to investigate the proportion of women who refrain from seeking medical treatment.

**Methods:**

A cross-sectional study was conducted with 1,214 Portuguese women aged 40–65 (*M*_age_=49.97; *SD*_age_=6.737), actively employed and self-reporting occasional/frequent urine loss. All data analyses were done with IBM SPSS Statistics and IBM SPSS AMOS. Structural equation modeling (SEM) was performed to analyze associations while adjusting for confounders such as age, education, menopausal status, BMI, and perceived sleep quality.

**Results:**

The predictive model showed an acceptable fit (CFI = 0.921; TLI = 0.850; RMSEA = 0.076; SRMR = 0.049). Higher UI symptom severity significantly predicted greater WP impairment (β = 0.440; *p* <.001). Age (β= − 0.107; *p* =.002) and education (β= − 0.061; *p* =.020) were associated with a lower impact of UI on WP, while poor sleep quality was associated with greater WP impairment (β = 0.121; *p* =.006). Notably, 60% of participants experiencing had not contacted a doctor regarding their symptoms, and 72.7% had never undergone treatment for UI.

**Conclusion:**

UI symptom severity negatively impacts WP, yet most affected women do not seek treatment. More effective workplace-based online interventions and accessible UI management strategies are essential to mitigating these effects. Future research should also incorporate objective clinical assessments and explore interventions tailored to different UI subtypes.

## Introduction

According to the International Continence Society (ICS), Urinary Incontinence (UI) is defined as the involuntary loss of urine, affecting approximately 30 to 40% of middle-aged women [1, 2]. Given its high prevalence, UI represents a significant public health concern.

However, only one in four women seek medical treatment, largely influenced by: (1) feelings of shame and social stigma, (2) dysfunctional UI beliefs (e.g., the misconception that UI is a minor problem and an inevitable long-term consequence of the natural aging process, pregnancy or postpartum), (3) limited awareness regarding available treatments, (4) low expectations of treatment efficiency, and (5) use of defensive and hiding strategies (e.g., avoid certain places, restrict social activities, drink less water); and (6) feeling able to self-manage [3, 4].

Several risk factors contribute to UI development, including obesity, aging, and menopause [5], since UI’s incidence is higher in middle-aged women, with a 30–40% prevalence [6]. Thus, the rationale for selecting the target population of the present study (40–65 years) stems not only from the increased prevalence of UI during this life stage, but also from the psychological changes associated with menopause, which can exacerbate UI symptoms due to estrogen depletion and weakened pelvic floor muscles [7].

Severity of UI symptoms is, in the vast majority of studies, correlated with the magnitude of its consequences [8]. Thus, UI symptom severity exerts a considerable impact on various aspects of these women’s lives, encompassing physical (e.g., less energy due to decreased sleep quality), psychological (e.g., low self-esteem), social (e.g., social isolation), sexual (e.g., decreased satisfaction), and economic repercussions (e.g., increased expenses on personal hygiene products) [9, 10]. Moreover, there might also be professional consequences associated with UI symptom severity, leading to a decline in Workplace Productivity (WP) [11].

The Organization for Economic Co-operation and Development (OECD) defines WP as an assessment of the percentage of time or number of days one has (or has not) been productive or functioning well while at work [12]. Hence, WP may be impacted by UI symptom severity, stemming from: (1) worries about interrupting work tasks with frequent trips to the bathroom, (2) concerns regarding work schedule (e.g., long hours without breaks) and location (e.g., distance from home), (3) diminished energy and ability to concentrate and engage in physical activities at work, (4) decreased self-confidence while working, (5) a higher likelihood of hospitals and pharmacies visits during work hours (leading to increased absenteeism), (6) and an elevated tendency to quit their jobs [13, 14].

Nevertheless, there remains a significant gap in the available literature regarding the impact of IU symptom severity on the WP. To address this research gap, further research is needed. Therefore, the present study aims to explore the impact of UI symptom severity on the WP of middle-aged women while controlling for relevant demographic and clinical variables. This objective was defined to answer the following research question: “Does UI symptom severity significantly predict WP impairment among middle-aged women?”. Additionally, this study seeks to assess the proportion of women with UI symptoms who refrain from seeking medical treatment.

It is hypothesized that higher UI symptom severity is associated with a more pronounced negative impact on WP (i.e., diminished WP), and that most women don’t contact their doctor regarding UI symptoms and don’t undergo any form of treatment. If supported, the findings of this study would underscore the economic and professional implications of UI symptom severity, highlighting the necessity for greater awareness, and for targeted and effective interventions within professional and workplace settings. To strengthen the reliability of our findings, potential confounders such as age, education level, menopausal status, body mass index (BMI), and perceived sleep quality were controlled.

## Materials and methods

### Design

The current study follows a cross-sectional, correlational and observational design.

### Participants

A total of 1,214 women, self-reporting occasional/frequent urine loss (*M*_age_=49.47, *SD*_age_=6.11), were selected (community non-probabilistic sample) through the dissemination of invitations via social media channels (i.e., particularly Facebook) and the distribution of a concise description of the research objectives among diverse groups of middle-aged/menopause-related women.

Inclusion criteria comprised: (1) age (40–65 years), (2) sex (women), (3) reports of occasional/frequent involuntary urine loss experiences when coughing or experiencing an urge to urinate (defined based on participant self-report using validated scales that measure urine loss frequency), (4) current employment status (active and full-time), and (5) internet access.

Exclusion criteria encompassed: (1) pregnancy or being within six months post-partum, (2) prior UI-related surgery; (3) abdominal, gynecological and breast cancer; (4) presence of neurological disorders; (5) and pelvic organ prolapse. Pelvic organ prolapse was initially screened via self-reported history of prior medical diagnosis and excluded due to its potential confounding effect on UI symptom severity; however, given the limitations of self-reporting, future research should consider alternative approaches for assessment. Table 1 presents participants characterization.

**Table 1:** Sociodemographic characteristics of participants (*N* = 1214)

	*N*	%
Age range 40–44 45–49 50–54 55–59 60–65	296 339 304 195 80	24.4 27.9 25.0 16.1 6.6
Nationality Portuguese Dual Nationality ^a^	1177 37	97.0 3.0
Level of Education 4th grade 9th grade 12th grade Bachelor’s degree Postgraduate degree Doctorate	2 83 336 472 290 31	0.2 6.8 27.7 38.9 23.9 2.6
Sexual-affective relationship Yes No	961 253	79.2 20.8
Number of biological children 0 1 2 3 >4	124 355 549 158 28	10.2 29.2 45.2 13.0 2.3

^a^ holds Portuguese and another nationality

### Measures

#### Sociodemographic and clinical characterization

Self-reported questionnaires were used to collect sociodemographic characteristics (e.g., age, education level, employment status, number of biological children), clinical details (e.g., obstetric history, diagnosis of physical and mental illnesses), and lifestyle factors (alcohol and tobacco consumption, intake of hot and cold beverages, high-impact physical activity). The data are presented in Table 2.

**Table 2:** Clinical characteristics and lifestyle habits of participants (*N* = 1214)

	*N*	%
Number of vaginal deliveries 0 1 2 3 >4	352 345 396 106 15	29.0 28.4 32.6 8.7 1.2
Number of caesarean deliveries 0 1 2 3 4	829 262 96 25 2	68.3 21.6 7.9 2.1 0.2
*Physical illness diagnosis* Yes No Asthma Hypothyroidism Diabetes Hypertension	379 835 48 35 30 69	31.2 68.8 4.0 2.9 2.5 5.7
*Mental illness diagnosis* Yes No Depressive disorder Anxiety disorder Obsessive disorder Psychotic disorder	165 1049 102 54 3 5	13.6 86.4 8.4 4.4 0.2 0.4
*Menopausal status* Premenopause Perimenopause Postmenopause	303 452 459	25.0 37.2 37.8
*Coffee intake* Yes No	1028 186	84.7 15.3
*Hot/cold beverages intake* Yes No	1033 181	85.1 14.9
*High impact physical activity* Yes No	175 1039	14.4 85.6
*BMI status* Underweight Healthy weight Overweight Obesity level I Obesity level II Obesity level III	28 507 443 178 44 14	2.3 41.8 36.5 14.7 3.6 1.2

Note. BMI, Body Mass Index

#### Exogenous variable – UI symptom severity

*Five items from King´s Health Questionnaire Symptom Severity Scale* [15]. Consists of a separate symptom checklist, derived from the King´s Health Questionnaire (KHQ) – translated and validated for European Portuguese [16]. This scale aims to measure the severity of Urinary Symptoms by assessing responses to specific items: 22 (“Frequency: going to the toilet very often”), 23 (“Nocturia: getting up at night to pass urine”), 25 (“Urge Incontinence: urinary leakage associated with a strong desire to pass urine”), 26 (“Stress Incontinence: urinary leakage with physical, e.g., coughing, running”) and 29 (“Waterworks infections”). Participants provide responses on a Likert scale ranging from 1 (“No at all”) to 4 (“A lot”). The global score is calculated by summing these items, yielding scores between 5 and 20 points, with higher scores indicating a higher UI symptom severity. Total scores below the midpoint (i.e., 12.5) indicated mild to moderate UI symptom severity, whereas total scores exceeding the midpoint indicated moderate to severe UI symptom severity. The psychometric properties of the scale were confirmed in this study, ensuring validity in the local language (i.e., Portuguese).

#### Endogenous variable – UI symptoms impact on the workplace productivity

*One item from Work Productivity and Activity Impairment Questionnaire (WPAI)* [17, 18]. This item measures the impact of IU symptoms on the Workplace (WP) by assessing participants’ responses to the question: “During the past seven days, how much did your UI symptoms affected your productivity while you were working?”, using a 10 point-Likert scale, with higher scores denoting a higher impact on WP. Only one item was used from the WPAI, since this was the only item specifically measuring productivity. Being used as a manifest variable, only the psychometric sensibility was analyzed, which revealed no severe violations of normality and displayed adequate skewness and kurtosis values for further parametric analysis (*Sk* = 2.04; *Ku* = 3.74).

#### Endogenous variable – perceived sleep quality

*One item from King’s Health Questionnaire* (KHQ) [16]. This item measures the participant’s perceived sleep quality through their responses to the question: “Does your bladder problem affect your sleep?”, using a four-point Likert scale, with higher scores denoting a higher impact on sleep quality. Being used as a co-variable, only the psychometric sensibility was analyzed, which revealed no severe violations of normality and displayed adequate skewness and kurtosis values (*Sk* = 1.40; *Ku* = 1.62).

### Sample size estimation

Before data collection, a minimum sample size of 10 participants per survey item was defined, as recommended for studies that aim to conduct confirmatory factor analyses (CFA) [19]. With a total of seven items, a sample size of 70 participants was required. Our final sample (*N* = 1,214) exceeded this threshold, enhancing the robustness of the CFA.

###  Data collection

The study´s data was collected online using the survey platform Google Forms, encompassing all the previously mentioned material.

#### Ethics issue

This study is part of the PURI-PRO’s (Portuguese Urinary Incontinence Project) research project. This project was approved by the ISPA-Instituto Universitário Ethical Committee for Research and followed the ethical guidelines and standards outlined by the Portuguese Psychologists Association (OPP) and the American Medical Association (AMA). All participants were required to read and agree to an informed consent statement, in which it was indicated that all ethical guidelines and standards were to be respected, assuring that participation was voluntary and anonymous. Contact information for the primary researcher was provided for any queries or concerns.

### Data analysis

All data analyses were conducted using the IBM SPSS Statistics (Statistical Package for Social Sciences) (v. 28), and the IBM SPSS AMOS (Analysis of Moment Structures) (v. 28).

Confirmatory Factor Analyses (CFA) were conducted to evaluate the psychometric properties of all employed instruments in this study. The model goodness-of-fit was assessed using the Comparative Fit Index (CFI), the Root Mean Square Error of Approximation (RMSEA), the Standardized Root Mean Square Residual (SRMR), and the Tucker-Lewis Index (TLI) [20]. CFI and TLI above 0.9, as well as SRMR and RMSEA below 0.08, were indicative of good model fit [21]. Convergent validity was used to determine the extent of association between the observed variables and was analyzed using the average variance extracted (AVE) of the factors, where values greater than 0.5 indicate adequate convergent validity [21]. Composite Reliability (CR) was used to assess reliability and values ​​equal to or exceeding 0.7 were considered acceptable indicators of reliability [21].

The following assumptions were also verified: normality of the data, absence of outliers, absence of missing values ​​and lack of multicollinearity between the exogenous variables [21, 22].

A Predictive Structural Equation Model was performed to study the hypothetical association between the variables under study (i.e., evaluate the statistical significance and the trajectory significance of the impact of UI symptom severity on the WP) [21].

## Results

### Clinical characterization

Regarding UI symptom severity, 609 (50.2%) women experienced mild symptoms, 521 (42.9%) reported moderate symptoms and 84 (6.9%) indicated severe symptoms. Furthermore, 728 (60%) women reported not having contacted their doctor about their UI symptoms and 883 (72.7%) did not undergo any form of treatment for UI.

### Main results

In the present study, the King’s Health Questionnaire Symptom Severity Scale presented evidence of good psychometric properties (reliability, sensitivity, and construct validity – factorial, convergent and discriminant validity).

**Fig. 1:**
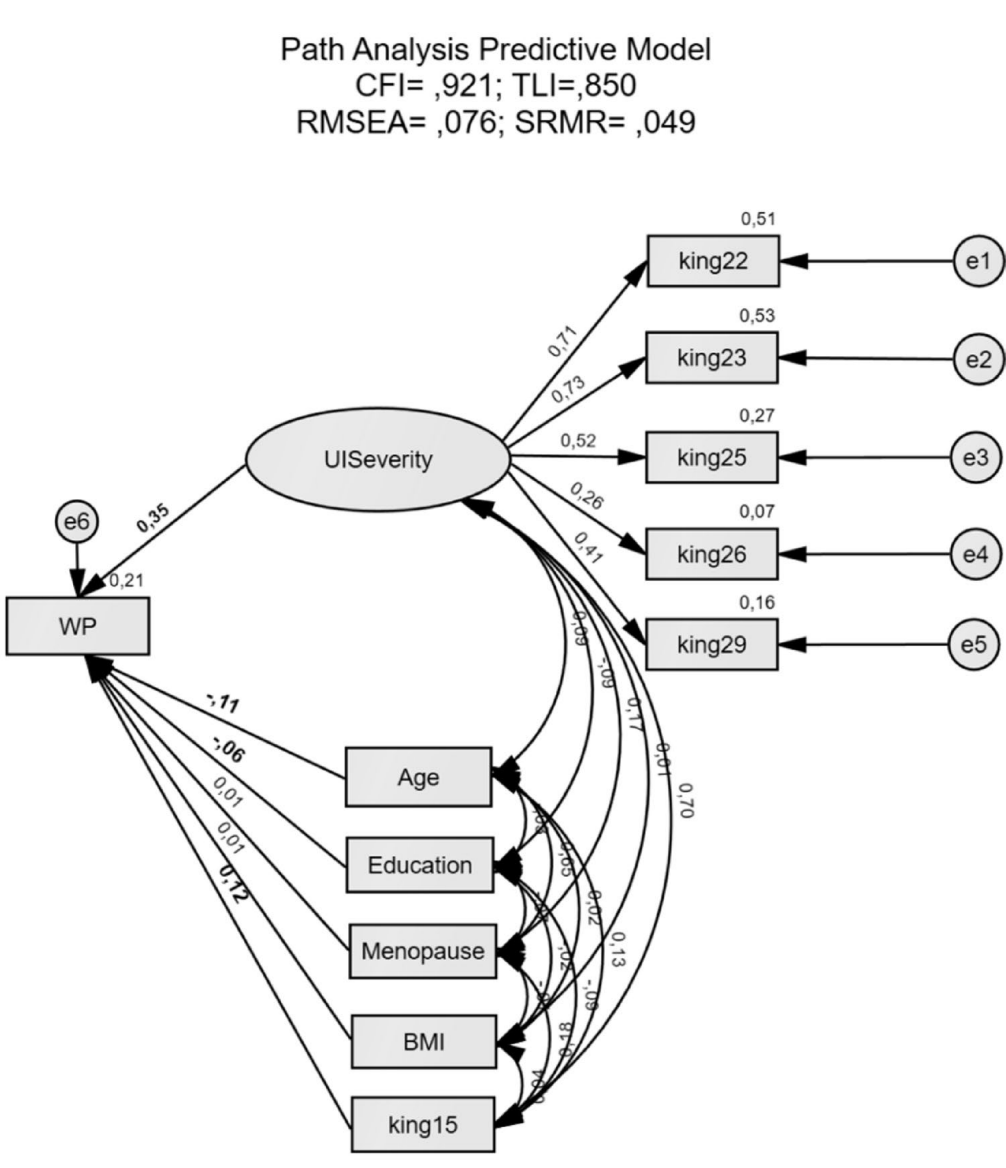
SEM model – predictive model. This SEM model shows UI symptom severity, age, education and perceived sleep quality (king15) as possible predictors of a greater impact on WP. The coefficients presented are standardized linear regression coefficients. Significant trajectories’ values are in bold. Note. UI = Urinary Incontinence, WP = Workplace Productivity, BMI = Body Mass Index

The SEM model (Fig. 1) presented an acceptable fit (CFI = 0.921; TLI = 0.850; RMSEA = 0.076; SRMR = 0.049). Regarding the trajectory significance, UI symptom severity emerged as a significant predictor of these symptoms’ impact on WP (β = 0.353; *p* <.001) (i.e., higher UI symptom severity was associated with a greater impact on WP). Overall, the model explained 21% of the variance of the impact on WP.

Regarding the co-variables’ trajectory significance, Age emerged as a significant predictor of UI symptoms’ impact on WP (β= − 0.107; *p* =.002) (i.e., a higher age was associated with a lower impact of UI symptoms on WP). Education also emerged as a significant predictor of UI symptoms’ impact on WP (β= − 0.061; *p* =.020) (i.e., a lower education level was associated with a high impact of UI symptoms on WP). Finally, perceived sleep quality also emerged as a significant predictor of UI symptoms’ impact on WP (β = 0.121; *p* =.006) (i.e., a higher UI symptom’s impact on sleep quality was associated with a greater impact of UI symptoms on WP). However, menopausal status (β = 0.009; *p* =.802) and BMI level (β = 0.010; *p* =.709) were not predictors of UI symptoms’ impact on WP.

## Discussion

The current study aimed to explore the impact of UI symptom severity on the WP of middle-aged women while controlling for relevant demographic and clinical variables, such as age, education level, menopausal status, BMI, and perceived sleep quality. Additionally, this study sought to assess the proportion of women with UI symptoms who refrain from seeking medical treatment.

Regarding the present study results, UI symptom severity was identified as a substantial predictor of WP impairment on middle-aged women. This finding suggests that women experiencing more severe UI symptoms are likely to face a greater negative influence on their WP.

These results are consistent with previous research, which has established a relationship between higher UI symptom severity and increased adverse effects on WP.^14^ Hence, these adverse impacts may arise from more frequent trips to the bathroom and visits to hospitals and pharmacies during working hours, diminished self-confidence and ability to concentrate while working, and even an increased tendency to quit their jobs [13, 14].

Age also emerged as a significant predictor of the impact of UI symptom severity on WP, indicating that older middle-aged women experience less pronounced negative effects of UI symptoms on WP compared to their younger counterparts. These findings contradict earlier studies, which have shown that UI symptoms generally become more severe with advancing age [23]. However, this discrepancy can be attributed to the fact that 1,130 participants (93.1%) in the current study reported only mild to moderate UI symptoms.

Furthermore, education attainment was found to be a significant predictor of the impact of UI symptom severity on WP, with women holding lower levels of education experiencing more pronounced negative effects. This aligns with prior research suggesting that women with lower educational levels often have limited awareness of pelvic health, including risk factors for UI and preventive strategies [24].

Perceived sleep quality also emerged as a substantial predictor of the impact of UI symptom severity on WP, implying that women who experience greater disruption to their sleep due to UI symptoms report a more significant negative effect on their WP. These findings corroborate existing research linking nocturia (i.e., getting up at night to urinate) with greater work impairment, including increased absenteeism and presenteeism [25].

Conversely, menopausal status and BMI did not emerge as significant predictors of the impact of UI symptom severity on WP. These results may be explained by the fact that the majority of participants (62.8%) were either in the premenopausal (i.e., reproductive stage preceding menopause) or postmenopausal (i.e., reproductive stage following menopause) phases, during which UI symptoms are generally less severe [26]. Additionally, only 19.5% of participants were classified as obese, a condition associated with greater urine loss.

Moreover, this study revealed that 60% of women experiencing UI symptoms reported not having contacted their doctor regarding these symptoms and 72.7% did not undergo any form of treatment. These results corroborate what has been widely documented in previous research, indicating that women experiencing UI symptoms often refrain from reporting them to their doctors [3, 4], underscoring the need for greater awareness and intervention strategies.

Regarding the moderated explanatory power observed in our model, one plausible explanation may be the exclusion of occupational characteristics intrinsic to specific professional roles. Certain job demands—such as prolonged standing, restricted restroom access, toxin exposure, occupational stress, shift work, long-distance travel, and sustained sedentary behavior—are often embedded in particular occupations and have been linked to the onset or aggravation of UI [27].

Furthermore, addressing UI subtypes in research may have important implications for occupational functioning and productivity. In particular, urgency and mixed U are typically more severe and characterized by sudden, uncontrollable urges to urinate, often resulting in frequent accidents and heightened embarrassment. This unpredictability can lead to psychological distress, increased healthcare utilization, and challenges in sustaining consistent job performance. Women with these subtypes report significantly more work-loss days due to medical absenteeism, higher rates of sick leave and short-term disability, and elevated absence-related costs for employers [28, 29].

In this context, workplace interventions addressing UI, besides bringing UI awareness, may play a crucial role in symptom mitigation by addressing occupational demands through lifting restrictions, scheduled breaks, ergonomic postural adjustments, enhanced restroom accessibility, and flexible time management strategies.

### Limitations

While this study offers valuable insights, several methodological limitations must be considered in interpreting its findings. These limitations arise primarily from the reliance on self-report measures, the digital mode of data collection, and the cross-sectional design employed.

First, the use of self-reported UI symptoms without clinical verification introduces a risk of reporting bias. Given the stigmatized and sensitive nature of UI, participants may underreport or misrepresent symptom severity, which could compromise the validity of symptom classification and obscure associations with psychological and occupational variables. Second, the exclusive use of online data collection limited participation to individuals with consistent internet access and digital literacy, thereby introducing potential socioeconomic sampling bias and reducing sample representativeness. The asynchronous nature of this methodology also restricted opportunities for clarification during the protocol, possibly affecting the quality and interpretability of participant responses.

Finally, the cross-sectional design precludes causal inference. Although associations between UI severity and work-related outcomes were identified, the temporal dynamics of these relationships remain unclear.

### Strengths

Despite these limitations, these findings suggest some theoretical and practical implications, alongside notable strengths. Firstly, the sample size facilitates a more comprehensive assessment of the variables under study. Secondly, we were able to confirm the considerable impact of UI symptom severity on WP (given that there is lack of research within this particular topic). Lastly, we used an instrument to measure UI symptom severity that considered not only frequency and quantity of urine leaks (as it has been done previously), but also incorporated items associated to nocturia, waterworks infections, stress and urge UI that allowed a more thorough assessment of UI symptom severity.

### Future research

To strengthen the validity and applicability of findings, future studies should consider implementing face-to-face data collection protocols. This would allow for real-time clarification of participant queries and improve data accuracy. In addition to self-report instruments, incorporating objective clinical assessments, such as physician-confirmed diagnoses or urodynamic evaluations, would enhance diagnostic precision and reduce the risk of reporting bias. Furthermore, integrating occupational risk factors/professional role and UI subtype-specific variables into future models may improve explanatory power and inform more targeted, effective interventions to support urinary health and workplace productivity.

To advance causal understanding, future research should adopt prospective longitudinal cohort designs, enabling the examination of how changes in UI symptoms influence occupational functioning over time. Such designs are critical for elucidating the dynamic interplay between UI severity, psychosocial well-being, and work capacity. Expanding recruitment beyond online platforms—through clinical, occupational, and community-based channels—will also improve sample diversity and external validity, allowing for a more comprehensive representation of UI experiences across sociodemographic strata.

By accounting for UI subtype in both research predictive modeling and intervention development, researchers and clinicians can more precisely address the occupational consequences of UI and implement strategies that mitigate productivity loss and enhance quality of life.

## Conclusion

The findings gleaned from this study hold potential relevance in fostering the development of and more targeted effective interventions, with multidisciplinary teams (e.g., psychologists, urologists, urogynecologists), addressing UI symptom severity to enhance WP among middle-aged women. Furthermore, given that a substantial proportion of participants reported not seeking medical attention for their UI symptoms, workplace initiatives – such as online educational programs and cognitive-behavioral strategies – geared towards raising awareness about the advantages of early intervention in UI and offering the possibility of contact with professional help if desired, may serve as practical solutions and as a catalyst for bolstering WP.

## Data Availability

The data supporting the conclusions of this article will be made available by the authors upon request.
